# Effects of Controlled Atmosphere on the Storage Quality and Aroma Compounds of Lemon Fruits Using the Designed Automatic Control Apparatus

**DOI:** 10.1155/2019/6917147

**Published:** 2019-06-17

**Authors:** Yuan Ma, Shaohua Li, Xiaocui Yin, Yage Xing, Hongbin Lin, Qinglian Xu, Xiufang Bi, Cunkun Chen

**Affiliations:** ^1^Key Laboratory of Grain and Oil Processing and Food Safety of Sichuan Province, College of Food and Bioengineering, Xihua University, Chengdu 610039, China; ^2^Administration for Market Regulation of Weiyuan County, Neijiang 642450, China; ^3^Key Laboratory of Food Non-Thermal Processing, Engineering Technology Research Center of Food Non-Thermal Processing, Yibin Xihua University Research Institute, Yibin 644004, China; ^4^College of Light Industry and Food Engineering, Sichuan University, Chengdu 610065, China; ^5^Key Laboratory of Physiological and Storage of Postharvest Agricultural Products of Agriculture Ministry, National Engineering Technology Research Center for Preservation of Agricultural Products, Tianjin 300384, China

## Abstract

‘Eureka' lemon fruits were stored under four controlled atmosphere- (CA-) combinations at 8°C for 20 days to investigate the effects on weight loss (WL), total soluble solids (TSS), titratable acidity (TA), vitamin C (VC), total phenolic content (TPC), sodium carbonate-soluble pectin (SSP), malondialdehyde (MDA), and volatile compounds. Results showed that the contents of TSS, TA, VC, and SSP in the stored fruits reduced during the storage period, while the WL and MDA increased. Fruit stored under CA2-combination (6 % O_2_+8 % CO_2_) showed the lower contents of WL and MDA and the higher content of TSS, TA, TPC, and VC than that of other treated fruits. The main volatile compounds present in the lemons were terpenoids, aldehydes, alcohols, and esters. In addition, both the terpenoid and aldehyde content are substantially higher in lemons exposed to CA2 conditions. In contrast, the alcohols and esters displayed elevated levels in the regular air (RA) stored fruit. In conclusion, CA with the suitable conditions proves to be better than RA as a storage regimen to keep the quality of lemons. These results indicated that the application of 6% O_2_+8% CO_2_ CA conditions could maintain the quality of ‘Eureka' lemon fruit during the storage time of 20 days and should be the optimal storage environment for postharvest Eureka lemons.

## 1. Introduction

The lemon fruits used in this study is classified as* Citrus limon* (L.) Burm. f, which is the third most important Citrus species after orange and mandarin. Lemons contain many high levels of both organic acids and health-promoting phytochemicals such as amines, vitamins, dietary fiber, lavonoids, and limonoids [1–3]. Moreover, citrus fruit is nonclimacteric fruit, with persistently low respiration and ethylene production rates [4]. However, peel pitting and peteca spots can occur on the lemons during storage [4, 5]. These disorders cause physiological and biochemical changes and eventually lead to fruit quality deterioration, such as VC loss, water loss, and decay during storage and transportation and cause massive commercial revenue losses [4, 5]. In recent years, the demand to develop appropriate operational techniques has increased significantly [6].

Among alternative treatments, the use of controlled atmosphere (CA) treatment has been extensively researched and the technology is commercially applied to several products, such as litchi fruits [7, 8], pomegranate [6], and apples [9, 10]. The technique of CA can provide different concentrations of gas, such as low O_2_ and high CO_2_ levels, which are always used with the appropriate temperature and relative humidity (RH) for fruits storage [6, 7]. Matityahu et al. (2016) had investigated the quality of different cultivars of pomegranate stored under CA (2 kPa O_2_ + 5 kPa CO_2_), wherein fruit quality of husks and aril juice was compared to that of fruit stored in air [6]. They also indicated that CA storage treatment was found to reduce the husk scald and decay development, albeit the RA could better maintain the anthocyanin level and preventing off-flavor development in aril juice [6]. Moreover, as reported by Martins and Resende (2015), the treatments of different CA combinations, such as 1% O_2_ and 12% CO_2_ and 3% O_2_ and 12% CO_2_, were found to minimize mass loss and fruit ripening [11]. Increased CO_2_ concentrations and reduced O_2_ levels in the storage atmosphere could reduce the respiration rate, delay the senescence, and thus extend the shelf life of fruits and vegetables, but might also induce adverse effects if the levels are either too high or too low [6].

Although MA packages can achieve a beneficial atmosphere at relatively lower cost compared to CA storage, their ability to create and maintain the optimum atmosphere conditions is limited due to the relatively narrow range and characteristic of gas permeability for plastic packaging materials [15, 16]. In CA storage apparatus, sophisticated mechanisms of O_2_ removal, CO_2_ production, and CO_2_ removal have been applied to control the atmosphere within a narrow target range at optimal storage temperature [15]. The fresh produce container system equipped with a gas diffusion tube can provide an automatically controlled environment with specifically measured O_2_ and CO_2_ concentrations and maintain the quality of spinach in a modified atmosphere environment at both constant and varying temperatures [15]. Although CA controlled environments are regularly applied to various fruits and vegetables, limited studies had been reported to employ the automatic CA equipment for keeping the storage quality of lemon fruit.

Therefore, the aim in this works was to investigate the influences of different concentrations of O_2_ and CO_2_ on the quality attributes of ‘Eureka' lemon fruits using the designed automatic CA control apparatus. First, the intelligent CA apparatus was introduced in order to provide the reference for other researchers. Then, the quality indexes including WL, TSS, TA, VC, TPC, SSP, MDA, and volatile compounds of lemon fruits under four CA combinations at 8°C for 20 days were evaluated.

## 2. Materials and Methods

### 2.1. Materials

Fruits belonging to lemon cv.* Eureka* were obtained from commercial growers from Anyue, a city in Sichuan province, China. The fruits were immediately transported on paved roads from the collection site to the laboratory in a ventilated vehicle. Sodium hydroxide, oxalic acid, sodium bicarbonate, 2-thiobarbituric acid (TBA), sulphuric acid (H_2_SO_4_), absolute ethyl alcohol, trichloroacetic acid (TDA), trans-1,2-diaminocyclohexane-N,N,N′,N′-tetraacetic acid (CDTA), glacial acetic acid, 2,6-dichloroindophenol indophenol sodium salt, and L-ascorbic acid were purchased from the Chengdu Kelon Chemical Reagent Factory (Chengdu, China).

### 2.2. Storage Conditions and Treatments

A total of 240 lemons were divided into four CA treatment groups: CA1=4 % O_2_+5 % CO_2_, CA2=6 % O_2_+8 % CO_2_, CA3 = 8 % O_2_+11 % CO_2_, and CA4=10 % O_2_+14 % CO_2_. A random selection of 60 lemons, uniform in size and appearance, were stored in an RA (21 % O_2_+0.03 % CO_2_) environment. Fruits subject to all treatments were stored for 20 days at 8°C and 85 %~90 % RH. Changes in the sample groups were documented every five days. Firstly, for each treatment, three fruits per replicate were peeled. The peel and pulp were immediately frozen with liquid nitrogen, freeze-dried, pulverized, and stored at –80°C until it was subsequently used to determine the TPC. Secondly, the same three fruits in each treatment were used to measure the WL, while four additional fruits were randomly selected to detect the TSS, TA, VC, and MDA content, respectively.

### 2.3. Determination of WL and TSS

An analytical balance was employed to monitor the individual weight of each lemon during their time in storage. The WL of each lemon was determined by utilizing a method identified by previous research [7]. In addition, the WL was calculated using the following equation and was expressed as a percentage.

(1) $$\begin{array}{c} W\left(\;\%\;\right)=\frac{m_{i}-m_{t}}{m_{i}}\times 100\% \end{array}$$

W represents the WL (%), m_i_ = initial fruit weight before storage (g), and m_t_ = final fruit weight after storage (g). The TSS were determined by using a method established in a similar study [17] with slight modifications. Lemon juice was extracted with an electric juicer and filtered through four cotton cloths until no juice remained. This process was followed by determining the TSS at an ambient temperature of 25 ± 1°C by using an automatic refraction meter (A610, Hanon Equipment Co., Jinan, China).

### 2.4. Determination of TA and VC

The TA was determined according to the method described in previous research [18] with slight modifications. Fresh fruit pulp was homogenized in a blender and filtered. A 10 mL volume of the extracted juice was diluted in distilled water to reach a quantity of 100 mL. The solution was transferred into a 250 mL beaker and placed over a magnetic stirrer to provide continuous motion to the sample solution. The juice was then titrated using standardized 0.1 mol/L NaOH at the phenolphthalein endpoint (pH = 8.2 ± 0.1). The TA was expressed as a percentage of the citric acid. The VC was determined by the 2, 6-dichloroindophenol titrimetric method as described in similar research [18]. The fruit pulp was homogenized in a Waring blender and filtered using a muslin cloth. Then, 5 mL of the clear juice was diluted to 50 mL using a met-phosphoric acid-acetic acid solution. A 7 mL quantity of this mixture was titrated against a standard indophenol solution. All extractions and titrations were performed in triplicate and the results were expressed in *μ*g ascorbic acid mL^−1^ pulp juice.

### 2.5. Pectin Extraction and Determination

Lemon peel and pulp were extracted using water to reduce the free sugar content before pectin was obtained [19]. Pectin was acquired from the water-extracted peel at optimal conditions of pH=1.8 and 85°C, for 30 min, and precipitated with isopropanol, according to a method reported in a previous study [20]. Furthermore, the supernatants obtained from the CDTA and Na_2_CO_3_ treatments were combined as SSP [21]. The sample was allowed to decompose entirely in 96 % H_2_SO_4_ containing disodium tetraborate (Na_2_B_4_O_7_·10H_2_O), followed by transformation into furfural derivatives. These secondary components reacted with the 3-phenylphenol to form a chromogenic product absorption at 530 nm. Galacturonic acid solutions (10-100 *μ*g/mL) were used as a standard calibration curve.

### 2.6. Determination of TPC

An adapted Folin-Ciocalteu method [18] was used for the determination of the TPC in the lemon peel extracts. A fresh 3.0 g sample of fruit tissue was homogenized with 30 mL of 60 % ethanol and centrifuged at 15000* g* for 5 min at 4°C. A 10 mL supernatants were diluted with 40 mL of 60 % ethanol for the next measurement. A 0.125 mL quantity of the diluted extract was mixed with 0.625 mL of distilled water, followed by the addition of 0.125 mL of Folin-Ciocalteu reagent. After 3 min, 1.25 mL of 7 % Na_2_CO_3_ and 1 mL of distilled water were added to the mixture. The solution was allowed a reaction time of 90 min in a darkened environment, followed by the measurement of the absorbance at 760 nm employing a spectrophotometer (UV726, T6, PG General, Beijing, China). A standard curve for gallic acid was used to quantify the TPC. The results were expressed as gallic acid equivalents per kg of fresh weight (FW) (mg·kg^−1^).

### 2.7. Determination of MDA

The MDA content was determined according to the method reported by Xing et al. [22] with slight modifications. Tissue (0.2 g) from the fruit slices were homogenized with 3 mL of 10 % TDA and centrifuged for 10 min at 10000* g*. This process was followed by mixing 2 mL of the supernatant with 2 mL of 6.7 g/L TBA (previously dissolved in 10 %). The obtained reaction solution was heat-treated for 30 min at 95°C, rapidly cooled in an ice bath and centrifuged at 10000* g* for 10 min to clarify the precipitation. The absorbance of the supernatant was measured at 450 nm, 532 nm, and 600 nm, respectively, by using a spectrophotometer (UV/VIS756-PC, T6, PG General, Beijing, China). The result was expressed as *μ*mol/g. FW.

(2) MDA content μmol/g∙FW=6.452×OD532−OD600−0.559×OD450×VtFW×Vs

where V_t_ is volume of the extract solution (mL), V_s_ is volume of the extract solution contained in the reaction mixture solution (mL), and FW is mass of fresh sample (g).

### 2.8. Determination of Volatile Compounds by GC-MS

#### 2.8.1. Volatile Compounds Quantification

A qualitative analysis of the volatile compounds was performed using a GC-MS system (Agilent Technologies, Santa Clara, CA, USA) consisting of a gas chromatograph (7890 A) in conjunction with a quadrupole mass spectrometer (MSD 5975 C), as described by relevant research [23] with slight modifications. Initially, 2 g of whole fruit pulp was weighed and placed in a 10 mL vial, followed by the addition of 4 mL water. The mixture was thoroughly vortexed to ensure homogeneity agitated at 400 rpm and 60°C for 30 min before extraction occurred. Subsequently, a 65 *μ*m DVB/PDMS SPME fiber (Supelco, Bellefonte, PA, USA) was exposed to the sorption surface above the liquid level for 10 min to perform the analyses. The fiber was introduced into the GC injector where desorption was conducted at 250°C for 5 min, followed by the initiation and collection of data. An HP-5-MS fused silica capillary column (30 m×0.250 mm inside diameter, 0.25 *μ*m film thickness; Agilent, USA) was used to separate the volatiles. The temperature of the injector, GC-MS interface, and ion source was at 250°C, 280°C, and 230°C, respectively. Analyses were performed using helium as a carrier gas at a column flow of 1.0 mL/min, and the electron impact ionization was 70 eV. The following oven temperature program was used: the initial temperature of 40°C was incrementally increased by 3°C min^−1^ to 150°C, followed by progressive temperature augmentation of 5°C min^−1^ to 300°C, where it was maintained for 3 min. The data was collected at a scanning range of 35-350. The analytical process to obtain the volatile compounds from the lemons was repeated three times. The proportion of each compound was estimated dividing its mean area by the total area of the chromatogram and expressed as percentages.

#### 2.8.2. Volatile Compound Identification

The volatile compounds were identified by a GC–MS QP2010plus (Shimadzu Corporation, Kyoto, Japan), as described by Wang et al. [24] with some modifications to the method. Initially, 2 g of lemon peel (previously thawed at ambient temperature), 1 g of NaCl, and 1 *μ*L of cyclohexanone standard solution (0.95 mg) were transferred to a SPME extraction bottle (20 mL) and preheated in a water bath at 40°C for 15 min before the extraction process occurred. Subsequently, a DVB/CAR/PDMS fiber (Supelco, 50/30 *μ*m×20 mm) was exposed to the headspace above the liquid surface for 45 min at 40°C. To quantify the volatile compounds, the same described GC and MS chromatographic conditions were adopted. The mass spectra of each compound were compared with those available in the National Institute of Standards and Technology (NIST) library and by comparing the linear retention index (LRI) with those available in existing scientific literature.

### 2.9. Statistical Analyses

The experimental data were analyzed by SPSS/PC version 23.0 (SPSS Inc., Chicago, USA) with one-way analysis of variance (ANOVA) and principal component analysis (PCA). The results were reported as the mean ± SD and the differences were recognized as significant (*p*<0.05).

## 3. Results and Discussion

### 3.1. Design Introduction of the Intelligent CA Apparatus

**Figure 1:**
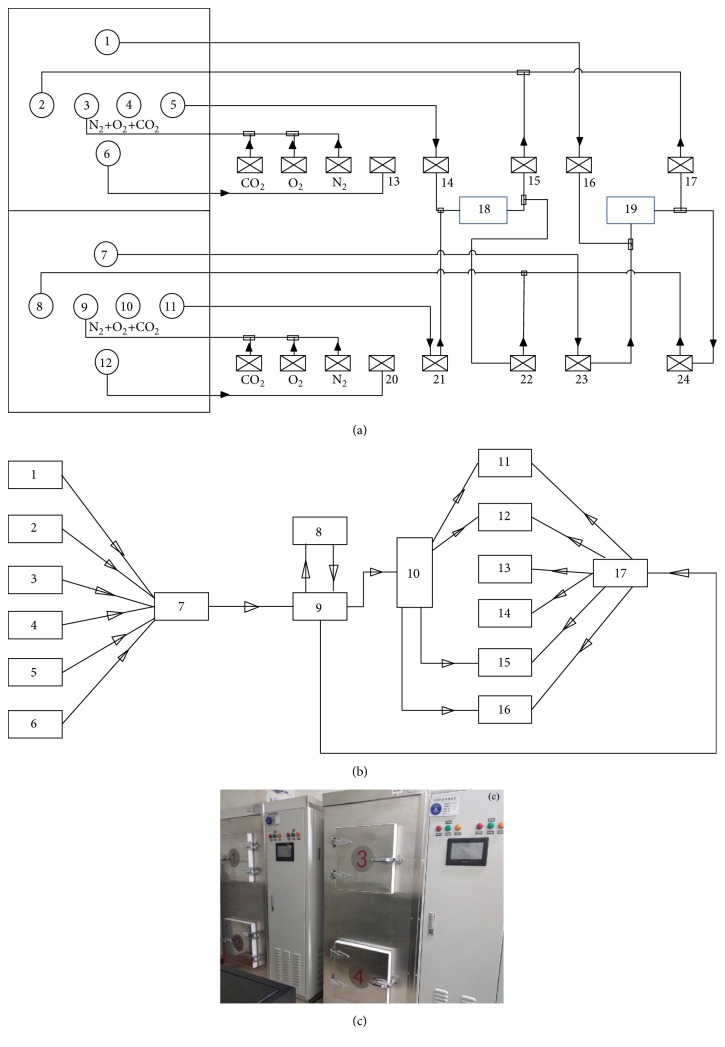
Layout diagram (a), control diagram (b) and experimental apparatus (c) of modified atmosphere controlled test box. ((a) where 1, 7, 16, 23: extracting ethylene; 2, 8: backing to gas; 3, 9: N_2_+O_2_+CO_2_; 4, 10: ozone; 5, 11, 14, 21: taking out the gas; 13, 20: exhausting gas; 15, 22: recovering gas; 17, 24: removing ethylene loop; 18: sensing components and pump; 19: machine of ethylene removal; (a) where 1: Temperature sensor; 2: oxygen sensor; 3: carbon dioxide sensor; 4: nitrogen sensor; 5: ethylene sensor; 6: humidity sensor; 7: analog module; 8: touch screen; 9: program mablelogic controller (PLC); 10: valve assembly; 11: nitrogen generator; 12: oxygen generator; 13: humidifier; 14: ozone sterilization machine; 15: CO_2_ gas source; 16: ethylene removal machine; 17: relay).

The CA equipment plays the crucial role for the storage of postharvested agriculture products. However, only few works were carried out on the improvement and design of the CA apparatus. In this study, the working principle and process of CA equipment used in this investigation are introduced in detail, as shown in Figures 1(a)–1(c), which might provide the reference for other researchers to develop the upgraded version of similar apparatus and for the investigation on the storage quality of other fresh fruits and vegetables. The configuration and control charts of CA equipment used in this work are provided and shown in Figures 1(a)–1(c). The model of an intelligent CA device used an automatic control system based on a Programmable Logic Controller (PLC). This device could detect and analyze the concentration of carbon dioxide and oxygen in the air, to regulate the gas composition within the CA chamber. As shown in Figure 1(a), the nitrogen in the air was modified to more than 99.5 % purity through an air compressor before it entered the CA chamber. Moreover, 99.9 % pure carbon dioxide was introduced to the chamber, followed by the addition of oxygen after purification and moisture removal. Subsequently, the automatic program was initialized to control the gas composition of oxygen and carbon dioxide to achieve the desired parameters (Figure 1(a)). Therefore, this device was successful in automatically removing the ethylene and controlling the RH.

As shown in Figure 1(b), the control chart consisted of six parts, including the sensors, the analog module, the PLC, the valve assembly, the relay, and six machines. The sensors comprise the nitrogen sensor, the oxygen sensor, the carbon dioxide sensor, the ethylene sensor, the temperature sensor, and the humidity sensor. Furthermore, the six machines refer to the nitrogen generator, the oxygenator, the humidifier, the ozone sterilizer, the CO_2_ source, and the ethylene removal machine. In this study, the lemon fruits were stored in a CA environment within 48 h of harvest, and both the CO_2_ and N_2_ in the CA chamber were obtained from the air compressor and membrane generator. The gas composition of the storage chamber was analyzed at 10 min intervals and adjusted when necessary. Generally, the level of O_2_ fluctuated between 4 % and 10 %, while the CO_2_ level was between 5 % and 14%, and that of RH was between 80% and 95%. Moreover, the CA storage regimes ranged from 5 d to 20 d. As shown in Figure 1(c), four CA chambers with a volume of 1 m^3^ were used to store the lemons and kept closed. A sensor was installed into each CA chamber to monitor the respective concentrations of O_2_ and CO_2_.

### 3.2. The Effects of CA and RA on the WL, TSS, and TA of the Lemons

**Figure 2:**
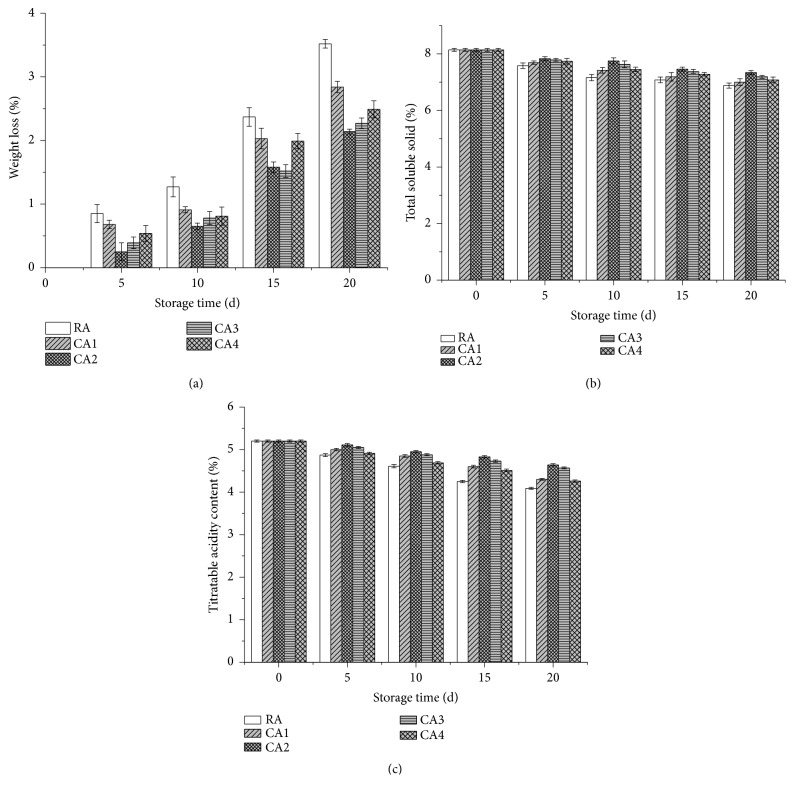
Effects of CA and RA on the WL, TSS and TA of lemons during 20 days' storage.

During the storage, the effect of CA and RA on the WL, TSS, and TA of the lemons was monitored (Figures 2(a)–2(c)). The WL index of the lemons exhibited rising tendencies with prolonged storage time (Figure 2(a)). The differences in the results obtained from the RA conditions were significant from day ten of the storage to the end of the experiment. At the conclusion of the storage time, the WL of fruit subject to RA, CA1, CA2, CA3, and CA4 were 3.52 %, 2.84 %, 2.14 %, 2.27 %, and 2.49 %, respectively. Exposing lemons to CA conditions significantly reduced the WL index. Moreover, CA2 with 6 % O_2_ +8 % CO_2_ exhibited a substantially lower WL index compared to other treatments (Figure 2(a)). On the one hand, the WL of all the samples increased with time, which was due to the matter consuming. Similar result was also found by other researchers. As reported by Ali et al. [7], fruit weight loss increased gradually with the extension of storage time. Moreover, according to the investigation of Selcuk and Erkan [25], the WL of ‘Istanbul' medlar fruit under modified and palliflex controlled atmosphere storage progressively increased with storage time and was linear for all treatments. On the other hand, the results obtained in this investigation also indicated that CA could maintain the weight of lemons more effectively than RA. Similar results were previously reported by some researchers [7, 25]. Selcuk and Erkan [25] reported that the WL of medlar fruit stored in MAP and CA was lower than that of an RA-stored sample. Ali et al. [7] also found that, among different CA combinations, litchi fruit stored under 1 % O_2_+5 % CO_2_ maintained substantially about 2.71-fold less weight loss during 35 days of storage [10]. Ali et al. [7] and Mendya et al. [26] demonstrated that the water is the main component found in fruits and vegetables, and the reduction of its loss from the commodity is the most critical requirement for maintenance of postharvest quality attributes. Moreover, the water loss of more than 4-6% (of the total fresh weight) results in visible wilting or wrinkling of the surface of most commodities [27, 28]. Respiration and transpiration are the main causes of weight loss in stored fruit and vegetables, which might induce the matter consuming [29, 30]. This is because that the stomata transpiration and direct evaporation through epidermal cell might induce the migration of the water from the fruit to the surrounding environment [31]. More importantly, weight loss was also significantly affected by storage conditions [16]. The low temperature, low content of O_2_, and high content of CO_2_ effectively inhibit the water migration and keep the final quality of stored fruits. As reported by Zhang et al. [32], the higher content of free water at strawberries cores retained better quality during storage. The weight loss of the control Ottomanit figs was extremely high, even though RH in the cold room was above 90%, probably reflecting the effects of advanced ripening and spoilage [7]. The reason for lower weight loss in CA-stored fruit can also be attributed to the slower ripening rate and respiration rate [33]. The suitable concentrations of O_2_ and CO_2_ in CA system might maintain the internal atmosphere of the fruit and are beneficial to the storage of lemon and have a greater effect on prolonging its storage period. Further studies focusing on the water migration, softening, and respiration rate of lemon during storage are necessary to investigate.

To further study the impact of storage conditions on the quality of lemons, the parameters commonly used to evaluate taste were measured, i.e., TA and TSS. As shown in Figure 2(b), when the storage time was increased, the TSS in fresh lemons displayed a decline in all relevant samples. At the end of the storage period, the lemons that were subject to a CA environment exhibited significantly higher TSS content than that in the RA-stored fruit. Additionally, the TSS in fruit treated with RA, CA1, CA2, CA3, and CA4 were 6.88 %, 7.00 %, 7.34 %, 7.19 %, and 7.08 %, respectively, while the marked retention of the TSS was obtained under the CA2 treatment at 6 % O_2_ + 8 % CO_2_. Similarly, Ali et al. [7] found that the soluble solid content (SSC) in litchi fruit continuously decreased during a 35-day storage period regardless of the treatment applied, while the highest retention level of the SSC was obtained during the CA treatment at 1 % O_2_ + 5 % CO_2_. In addition, similar results were obtained by Tian et al. [8], who reported the SSC decreased with prolonged storage time, and the CA was more effective than MAP (modified atmosphere packing) and RA treatments in maintaining the SSC of the fruits. Thus the CA-stored fruits maintained a higher TSS content that might be attributed to the suppressed senescence caused by the CA treatment [34, 35]. Furthermore, CA treatment could reduce the metabolic activity of the fruits that retarded the polysaccharide degradation reactions [36]. The CA consisting of reduced O_2_ and elevated CO_2_ concentrations inhibited the respiration rate and sugar depletion in the samples, thus maintaining the higher TSS level than RA treatment [6].

TA is commonly used to demonstrate the ripening stage of the fruits as well as evaluate the fruit taste which is represented mainly by the balance between sweetness and acidity [35]. The data analysis showed that the TA of fresh lemon juice decreased with prolonged storage time in all stored samples (Figure 2(c)). At the end of the storage period, the lemons subject to a CA environment exhibited higher TA content than that of the RA-stored fruit. The TA of fruit treated with RA, CA1, CA2, CA3, and CA4 were at 4.09 %, 4.30 %, 4.64 %, 4.57 %, and 4.26 %, respectively, while the marked retention of the TA was obtained from the CA2 treatment at 6 % O_2_ + 8 % CO_2_ (Figure 2(c)). Similarly, Ali et al. [7] found that the TA levels in litchi fruit significantly decreased during storage. Additionally, the fruit subject to 1 % O_2_ +5 % CO_2_ CA conditions maintained the highest TA content at the end of the experiment. Matityahu et al. [6] also reported the TA in pomegranates declined during storage. The reason for high TA in CA-stored fruit could also be attributed to the slower ripening rate and reduced respiration rate [27]. The concentration of O_2_ and CO_2_ played the crucial role in inducing the slower ripening rate of pear samples [37]. This result might also be due to the reduced decarboxylation of organic acids including citric acid and malic acid in fruit exposed to low O_2_ and high CO_2_ levels during storage [38], which are always used as the substrates for the enzymatic reactions of respiration [39]. The decrease of TA is also associated with cellular activity, in which organic acids serve as substrates that enter the Krebs cycle to gain energy for repairing the aging cells and membranes [40].

### 3.3. Effects of CA and RA on VC and TPC Levels in Lemons during Storage

**Figure 3:**
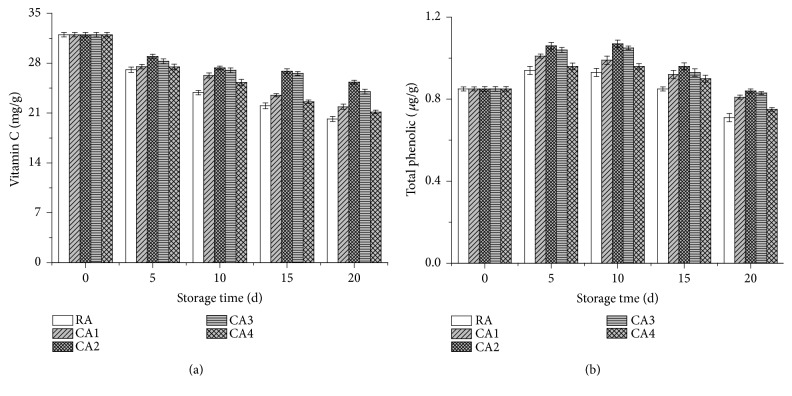
Effects of CA and RA on the VC and TPC of lemons during 20 days' storage.

Results regarding the impact of CA and RA conditions on VC and TPC levels are shown in Figures 3(a) and 3(b). As shown in Figure 3(a), during the period of storage, the VC content of the lemons gradually decreased irrespective of the treatments. However, averaged over the control, CA-stored fruit showed significantly higher VC content during the 20-day storage period. At the end of the storage time, the lemons subject to CA maintained higher VC content than that in the RA-stored fruit. The VC content of the fruit treated with RA, CA1, CA2, CA3, and CA4 were 20.17, 21.89, 25.34, 24.02, and 21.15 mg/g, respectively, while the marked retention of the VC was obtained during CA2 treatment at 6 % O_2_ + 8 % CO_2_ (Figure 3(a)). Similarly, studies performed by Selcuk and Erkan [25] suggested that a decreasing trend was present in the ascorbic acid levels of all stored medlar fruit throughout the 60-day storage period. Tian et al. [41] also found that the VC content of sweet cherries decreased with prolonged storage time and fruits subject to 5 % O_2_ + 10 % CO_2_ showed higher VC content than that in the MAP-stored and 70 % O_2_ + 0 % CO_2_ stored samples after 60 days of storage. The decrease of ascorbic acid under prolonged storage conditions might be due to the utilization of different organic acids during fruit respiration or their likely conversion to the sugars [7]. Higher retention of VC was observed in litchi fruit kept at CA_1_ (3% O_2_ +7% CO_2_) than the fruit in CA_2_ (17% O_2_ +6% CO_2_), and this was attributed to reduced enzymatic oxidation in an environment presenting low O_2_ and high CO_2_ conditions [33]. The ascorbic acid content reduced with advancement in fruit maturity, and the CA-stored samples exhibited higher retention of VC during 20 days storage period than the fruit in RA. However, this result might be attributed to the inhibited respiration rate and the reduced oxidation of various organic acids [7, 10].

The health benefits of lemons are related, at least in part, to its TPC. As shown in Figure 3(b), the TPC of the lemons increased in all treated samples during the first 5 days, followed by a gradual decrease over a prolonged storage period. On day 20, the lemons stored in CA maintained higher TPC than the RA-stored fruit. The TPC of fruit treated with RA, CA1, CA2, CA3, and CA4 were 0.71, 0.81, 0.84, 0.83, and 0.75*μ*g/g, respectively, while the marked retention of the TPC was obtained during the CA2 treatment at 6 % O_2_ + 8 % CO_2_ (Figure 3(b)). Similarly, Ali et al. [7] found that the TPC was significantly decreased during a 35-day storage period of litchi fruit. On day 35, the fruit subject to 1 % O_2_ +5 % CO_2_ CA conditions maintained the highest TPC level. Tian et al. [8] also found that the TPC of litchi fruits stored under CA conditions decreased to 42.0-43.8 *μ*g/g after 14 days. The reduction of TPC in stored lemon fruits can be attributed to the change in enzyme activity and the removal of astringency during the ripen process resulting in phenolic degradation [25, 42]. The high TPC level in the CA-stored samples might be due to the result of inhibited oxidation and decreased membrane leakage. The impaired membrane integrity ultimately might induce the peroxidase (POD) and polyphenol oxidase (PPO) to combine with the content [7].

### 3.4. The Effects of CA and RA on the SSP Content of Lemons during Storage

**Figure 4:**
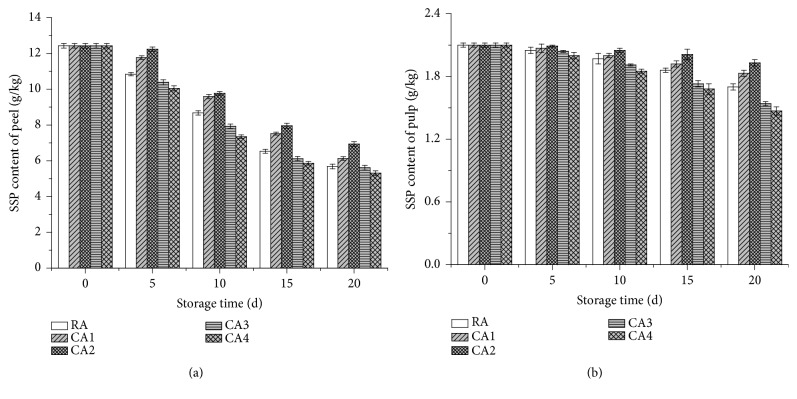
Effects of CA and RA on the SSP content of lemon peel and pulp during 20 days' storage.

The changes to the cell wall structures, especially pectin components are mostly correlated with the textural softening of fruit [29]. The SSP content in the lemon peel tissue gradually decreased with prolonged storage time irrespective of which treatments were applied (Figure 4(a)). On day 20, the SSP in the lemon peel treated with RA, CA1, CA2, CA3, and CA4 were 5.68, 6.13, 6.94, 5.61, and 5.31 g/kg, respectively, while the marked retention of the SSP was obtained during the CA2 treatment at 6 % O_2_ + 8 % CO_2_. Considering the SSP content of the pulp, a similar trend was observed, where a gradual decrease was evident in conjunction with an extended storage period (Figure 4(b)). Therefore, after 20 days of storage, the SSP in the lemon pulp treated with RA, CA1, CA2, CA3, and CA4 were 1.70, 1.83, 1.93, 1.54, and 1.47 g/kg, respectively, while the marked retention of the SSP in lemon pulp was obtained during the CA2 treatment at 6 % O_2_ + 8 % CO_2_ (Figure 4(b)).

Results indicated that the SSP content in the lemon peel tissue of all the samples decreased with prolonged storage time. This might be due to the change of water-insoluble protopectins into water-soluble pectins during the storage period, which could also induce the loss of fruit firmness [33, 43]. As demonstrated by Yang et al. [43], in the early storage time, the pectin consisted of linear single fractions, long chains, branch structure, short chains, and polymers might not be depolymerized by those pectinolytic enzymes [29, 43]. Furthermore, with extending the storage time, the degradation of pectin chain widths occurred, which was a natural senescence process of fruits as pectinolytic enzymes in fruits continued to degrade pectin [43, 44]. As indicated by Liu et al. [45], a larger number of branched structures and fractions with multiple branching structures were found in the untreated samples. Net-like structures were no longer observed at the end of storage [43]. On the other hand, the highest retention of the SSP was observed in the samples with CA2 treatment (6 % O_2_ + 8 % CO_2_). Similar results were obtained by previous studies [46, 47]. Yang et al. [46] found that the gradual degradation of SSP was observed in yellow peaches when exposed to prolonged storage time under CA conditions. Huyskens-Keil et al. [47] reported that after a 21-day storage period, the insoluble pectin content in mature Pepino fruits decreased, while the levels of water-soluble pectin (WSP) was partially prevented from increasing by the continuous and varying CA treatments. The reduction of pectin might be associated with various enzyme activities, such as pectin-methylesterase and polygalacturonase [47]. High CO_2_ concentrations were helpful in reducing softening in ripe Pepinos; this result could mainly be ascribed to the inhibition of the conversion of protopectin to soluble pectin by the suppression of degrading cell wall enzyme activity [47]. CA conditions can impede the degradation of the connections between chelate-soluble pectin molecules (CSP), causing a higher retention level of pectin as evident from the CA1 (2% O_2_ +10% CO_2_) and CA2 (5% O_2_ +5% CO_2_) treatments [48]. The fruit subject to CA2 treatment at 6 % O_2_ +8 % CO_2_ showed higher level of SSP that might be the result of the degradation of SSP molecules that were inhibited by concentrations containing lower O_2_ and higher CO_2_ levels [46]. The suitable CA treatment could inhibit the degradation of pectin chains by inhibiting the respiration and metabolism rate of fruits, thus reducing the activity of pectin degrading enzyme [30]. Moreover, the SSP chain width and nanostructural morphologies could also influence the fruit firmness [30]. On the other hand, the different CA conditions might also affect the solubilisation and depolymerization processes of CSP and the depolymerized and degraded processes of WSP side chains during fruit softening [44]. These functions could also induce the network structure of pectin [43]. However, the relationship between the changes in SSP content, the firmness of fruits during storage, the optional parameters, and technologies is still unclear and should be also considered. More importantly, the influencing mechanism of CA conditions on the pectin degradation in lemon fruits is also important. Further research works are necessarily conducted in order to understand these issues.

### 3.5. The Effects of CA and RA on the MDA in Lemons during Storage

**Figure 5:**
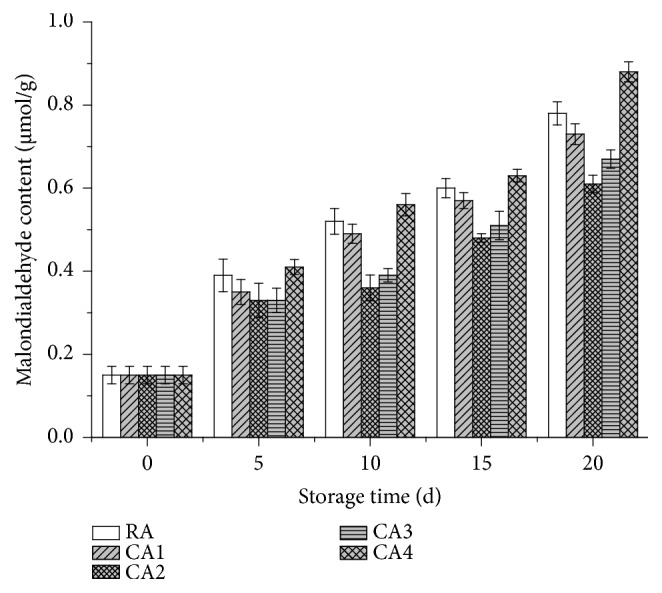
Effects of CA and RA on the MDA content of lemons during 20 days' storage.

MDA is the product of lipid peroxidation and is often used as direct indicators of membrane injury and cellular oxidative damage [22]. As shown in Figure 5, the continuously growing trends of MDA content were observed both in control and CA treated lemon fruit. On day 20, the MDA contents in lemon fruit treated with RA, CA1, CA2, CA3, and CA4 were 0.78, 0.73, 0.61, 0.67, and 0.88 *μ*mol/g, respectively. Additionally, the fruit that were subject to CA2 (6 % O_2_ +8 % CO_2_) conditions displayed a lower MDA (0.61 *μ*mol/g) content than those exposed to other storage conditions. This result was 0.17 *μ*mol/g lower than that obtained from the RA-stored sample. Given the various CA combinations, fruit stored in CA2 conditions exhibited a substantially lower MDA content during a 20-day storage period.

Similar results were previously reported by some researchers [7, 10]. Ali et al. [7] reported that the MDA content in all stored litchi fruit samples increased during a 35-day storage period. However, the fruits kept in 1 % O_2_ +5 % CO_2_ conditions displayed a lower MDA content (60 %) than the samples subject to other treatments. Studies performed by Mditshwa et al. [10] suggested that the MDA content in all stored apples (cv. Granny Smith) increased with prolonged storage time, which was caused by elevated lipid peroxidation. These results indicated that the MDA content is a significant lipid peroxidized product, which reflects the actual extent of membrane-lipid-peroxidation induced by reactive oxygen species (ROS) [7, 22]. Increased production of ROS leads to enhanced lipid peroxidation, causing membrane deterioration [7]. The lower MDA production in CA1, CA2, and CA3 stored fruit can be attributed to reduced ROS accumulation, therefore, inhibiting membrane peroxidation [7]. However, the CA4-stored sample had higher MDA content than those subject to other CA treatments. This might be associated with the high oxygen atmospheres that promote a higher level of lipid oxidation [49].

### 3.6. Multivariate Analysis-PCA

**Figure 6:**
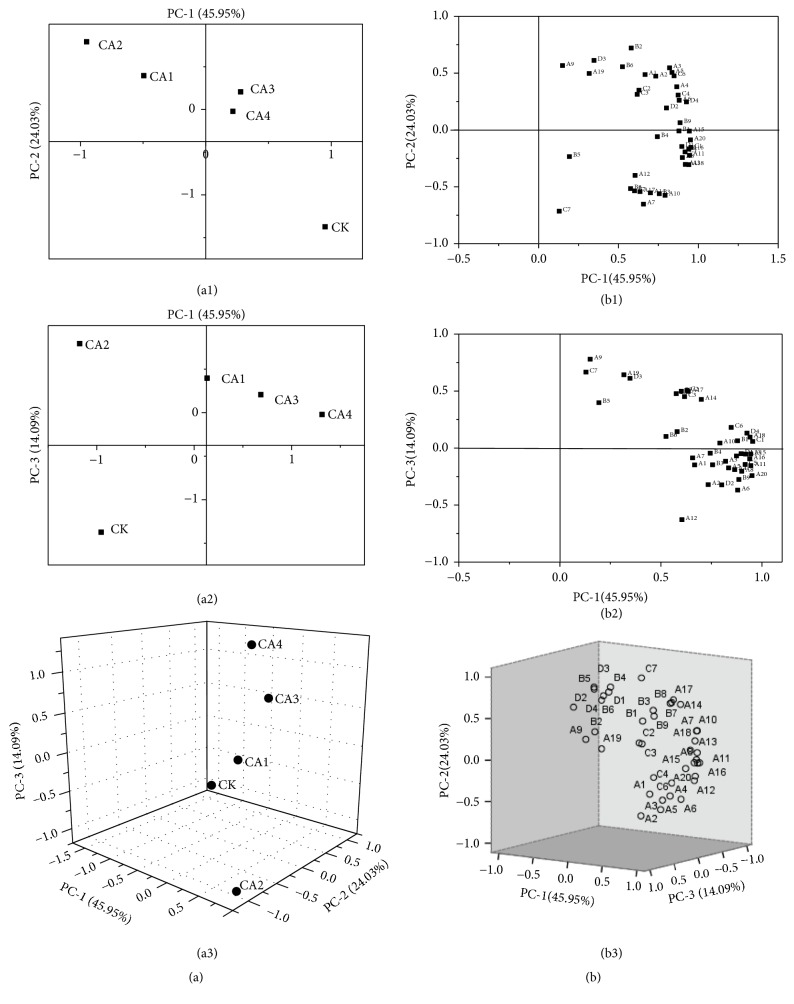
Principal component analysis (along PC-1and PC-2, PC-1, and PC-3) of the volatile profile of lemon fruit stored under control (CK), and different controlled atmosphere (CA) environments (CA1=4 % O_2_+5 % CO_2_, CA2=6 % O_2_+8 % CO_2_, CA3 = 8 % O_2_+11 % CO_2_, and CA4=10 % O_2_+14 % CO_2_) after 20 days of storage. (a) Scores plot; (b) loadings plot.

A PCA was conducted to obtain a broader view of the biochemical changes taking place in CA and RA-stored fruit. PCA scores and loadings are shown in Figure 6. Clear separation was observed demonstrating the effects of the storage conditions on the volatile components in Eureka lemons. The first three results account for 84.07 % (F1 = 45.95 %; F2 = 24.03 %; F3=14.09 %) of the total variance and clearly separated fruit stored in CA and RA environments, exhibiting two distinct clusters.  Figure 6(a) (scores plot) shows that the CA1 treated group had similar properties to the CA2 group, and the CA3 treated group had similar properties to the CA4 group. However, the control (CK) group had distinct properties from the other groups.  Figure 6(b) (loading graph) provides a comprehensive view of the relationships among the volatiles measured in this experiment. According to Liu et al. [50], the classification of factor loadings is considered strong, moderate, and weak when corresponding to loading values of 0.75, 0.75-0.50, and 0.50-0.30, respectively. As shown in Figure 6(a), all volatile components were positively correlated with PC1 (45.95 %). Therefore, the scores can be interpreted by factor loadings, with PC1 positively associated with terpenoids. Consequently, terpenoids have a greater impact on the volatile components of lemons. Terpinolene, (Z)-*α*-Bergamotene, *β*-Santalene, *β*-Caryophllene, Citronellal, Decanal, and Geranyl-propionate were considered as the main volatile compounds in lemons.

### 3.7. The Effects of CA and RA on the Aroma Compound Analysis of Lemons during Storage

A total of 40 volatile compounds were identified by the GC/MS in RA, and CA-stored lemons, and were grouped according to the following chemical classes (Table 1): terpenoids (20 compounds identified), alcohols (9), esters (4), and aldehydes (7). These components were detected in all samples following the conclusion of the storage period, regardless of the maturity levels of the fruits. In all samples, the terpenoids (935.7-1029.74 *μ*g/g) followed by aldehydes (68.39-116.4 *μ*g/g) were the main components. Terpenes and aldehydes were considered instrumental in the aroma and flavor of orange juice [51]. The results corresponded with those previously reported, wherein the terpenoids were described as the principal group present in herbivore-induced plant volatiles [52].

On day 20 of the storage process, fruits that were kept in a CA2 environment exhibited elevated levels of total terpenoids and aldehydes, while displaying a lower ester amount in comparison to other storage conditions (Table 1). However, fruit that were exposed to RA contained more moderate levels of terpenoids and aldehydes, while the esters and alcohols were significantly higher (Table 1). At the end of the storage period, the terpenoid content in lemons treated with RA, CA1, CA2, CA3, and CA4 were 935.70, 1009.60, 1029.74, 984.17, and 963.56*μ*g/g, respectively. Furthermore, fruit subject to CA2 (6% O_2_ +8% CO_2_) treatment displayed higher terpenoid content than samples subject to other treatments. A major terpenoid compound in lemons is limonene (723.36-741.60 *μ*g/g), followed by *γ*-Terpinene (115.56-132.00 *μ*g/g) (Table 1). The similar results were obtained by Zhong et al. [12], who reported that d-limonene was far higher than the other free volatile compound and was usually a main compound in lemon, and *γ*-Terpinene was the second major compound in Eureka lemon. Terpenoids accounted for the highest concentrations, about 10-, 25-, and 50-fold higher than those of aldehydes, alcohols, and esters, respectively (Table 1). Aldehydes are crucial ester-precursors, since they represent the first step in volatile compound formation [53]. Low levels of esters and alcohols were observed in CA-stored samples, indicating that these compounds were negatively affected by the CA environment.

Recently there are some related reports involving the impact of a CA on the volatile compounds in fruit [6, 54, 55]. Considering ‘Royal Gala' apples, research indicated that the emission of straight-chain esters decreased when exposed to extremely low O_2_ (0.5 kPa), which is related to the aroma [54]. However, studies performed by Matityahu et al. [6] suggested that the levels of ethanol, ethyl acetate, and acetaldehyde in pomegranates increased significantly during a 5-month storage period. Moreover, these volatiles appeared to be higher in a CA environment than when exposed to RA. Additionally, Defilippi et al. [55] reported that, depending on the specific compound, the levels of ethanol, acetaldehyde, and ethyl acetate were 3 to 12 times higher in CA-stored fruit than samples subject to RA. The difference between the research conducted for this paper and similar studies may be due to variations in determination assays and specific fruit cultivars. Results indicated that the CA-stored lemons are characterized by higher terpenoids and aldehydes, while the alcohols and esters remained at lower levels. This is an indication that treatment with a CA can effectively maintain the terpenoids and aldehydes, while negatively impacting the release of esters and alcohols. The CA treatment suppressed the production of ethylene and the rate of respiration. Therefore, since the biosynthesis of the volatile compounds is closely related to the ethylene presence and respiratory activity, the CA-stored fruits contained lower levels of esters and alcohols [9]. In addition, a CA environment characterized by low O_2_ levels and high CO_2_ levels successfully inhibited ethylene biosynthesis and activity. This process is crucial for ester forming enzymes to function correctly. Therefore, fruit stored in CA conditions displayed lower ester levels [9]. Although CA treatment negatively affects the ester and alcohol content in lemons, the fact that it efficiently maintains terpenoids, aldehydes, and other vital properties during long-term storage allows for a high-quality product that is acceptable to consumers [54, 56].

## 4. Conclusion

The suitable condition of CA system can provide the excellent control on the quality loss of stored fruits. In this works, CA storage of lemon under CA2 (6 % O_2_+8 % CO_2_) substantially maintained TSS, TA, VC, SSP, and TPC reduced WL and MDA contents. In regard to volatile compounds of lemon, the main volatile compounds consisted of terpenoids, aldehydes, alcohols, and esters. The CA-stored fruits contained lower levels of alcohols and esters, while displaying high retention of terpenoids and aldehydes. Results in this works indicated that the treatment of CA2 (6 % O_2_+8 % CO_2_) might provide the optimal storage conditions for the storage of ‘Eureka' lemon. Furthermore, the water migration and respiration rate can influence the fruit quality, especially in the longer storage period. The influencing mechanism of CA conditions on the pectin degradation in lemon fruits is also important. Further research works are necessary to conducted in order to understand these issues.

## Figures and Tables

**Table 1:** Effects of CA and RA on the volatile compounds of lemons.

No.	Name		Control	CA1	CA2	CA3	CA4	Flavour description
	Σ Terpenoids		935.70	1009.60	1029.74	984.17	963.56	
1	4-Carene^*∗∗∗*^	A1	4.20±0.13^^e^^	6.60±0.20^d^	11.40±0.15^a^	9.84±0.16^c^	10.56±0.17^b^	Lemon, Flower, Mint [12]
2	*α*-Thujene^*∗∗∗*^	A2	6.24±0.25^d^	14.16±0.21^a^	12.72±0.21^b^	11.64±0.23^c^	11.28±0.20^c^	Sour, Flower, Mint [12]
3	*α*-Pinene^*∗∗∗*^	A3	27.96±1.25^c^	40.48±1.01^b^	43.44±1.13^a^	43.44±1.24^a^	41.76±1.17^ab^	Sweet, Fruit, Grass Lemon [12, 13]
4	*β*-Pinene^*∗∗∗*^	A4	0.84±0.15^d^	2.04±0.15^a^	1.92±0.13^ab^	1.80±0.14^ab^	1.68±0.15^c^	Pine, Resin, Wood, Turpentine [12, 13]
5	*β*-Myrcene^*∗∗∗*^	A5	19.80±1.02^c^	35.24±1.24^ab^	36.00±1.59^a^	35.40±1.25^ab^	33.36±1.48^b^	Lemon, Orange, Resin [12]
6	*α*-Phellandrene^*∗∗∗*^	A6	1.20±0.20^d^	2.64±0.30^ab^	3.00±0.10^a^	2.52±0.20^bc^	2.16±0.15^c^	Lemon, Flower, Milk [12]
7	d-Limonene	A7	723.36±23.20^a^	738.00±21.45^a^	741.60±20.98^a^	724.56±20.58^a^	701.16±19.75^a^	Citrus, Mint, Lemon [12]
8	(Z)-*β*-Ocimene^*∗*^	A8	0.30±0.12^d^	0.56±0.12^ab^	0.60±0.11^a^	0.48±0.10^abc^	0.35±0.13^bc^	Fruit [12]
9	(E)-*β*-Ocimene^*∗∗∗*^	A9	3.00±0.20^d^	3.60±0.18^c^	2.52±0.15^e^	3.96±0.17^b^	4.68±0.16^a^	Fruit [12]
10	*γ*-Terpinene	A10	115.56±15.52^a^	124.92±14.29^a^	132.00±13.98^a^	112.80±15.03^a^	117.48±15.16^a^	Lemon, Flower, Oil, Anise, Mint [12, 13]
11	Terpinolene	A11	7.56±1.66^b^	10.32±1.32^ab^	11.64±1.71^a^	9.00±1.59^ab^	9.48±1.19^ab^	Resin, Citrus, Lemon [12]
12	(Z)-Sabinene^*∗∗∗*^	A12	1.56±0.15^bc^	1.80±0.18^b^	2.72±0.22^a^	1.44±0.14^d^	1.56±0.15^bc^	-
13	(Z)-*α*-Bergamotene	A13	0.48±0.10^a^	0.72±0.10^a^	0.78±0.20^a^	0.60±0.15^a^	0.60±0.18^a^	Lemon, Sour [12]
14	Caryophyllene	A14	7.32±2.54^a^	7.64±1.85^a^	7.80±2.35^a^	7.61±2.46^a^	7.29±2.27^a^	Clove [12]
15	(E)-*α*-Farbesene^*∗*^	A15	0.72±0.27^b^	1.08±0.10^a^	1.20±0.13^a^	0.96±0.11^b^	1.08±0.16^a^	-
16	*β*-Santalene	A16	0.36±0.10^b^	0.60±0.10^a^	0.60±0.10^a^	0.48±0.10^b^	0.48±0.10^b^	-
17	*β*-curcumene	A17	0.24±0.10^a^	0.24±0.18^a^	0.24±0.12^a^	0.24±0.15^a^	0.24±0.13^a^	-
18	*β*-Cayophllene	A18	0.48±0.14^a^	0.60±0.13^a^	0.72±0.12^a^	0.60±0.14^a^	0.60±0.15^a^	-
19	Elixene^*∗∗∗*^	A19	1.92±0.12^c^	2.40±0.12^b^	1.92±0.13^c^	2.28±0.14^b^	3.00±0.14^a^	-
20	(Z)-*α*-Bisabolene^*∗*^	A20	12.60±1.21^c^	15.96±1.13^ab^	16.92±1.14^a^	14.52±1.2^bc^	14.76±1.18^abc^	-

	Σ Alchols		47.49	24.48	40.28	42.95	41.68	
21	*β*-Linalool	B1	2.52±0.13^b^	2.40±0.10^b^	2.64±0.14^b^	2.76±0.12^a^	2.76±0.15^a^	Lemon, Sweet Fruit [12, 13]
22	terpinen-4-ol^*∗∗∗*^	B2	9.96±0.15^b^	3.24±0.13^e^	9.20±0.15^d^	9.60±0.14^c^	13.80±0.12^a^	-
23	(Z)-Verbenol	B3	0.12±0.06^a^	0.12±0.07^a^	0.12±0.05^a^	0.24±0.08^a^	0.12±0.05^a^	-
24	(Z)-Carveol^*∗∗*^	B4	0.33±0.01^ab^	0.24±0.03^c^	0.24±0.05^c^	0.35±0.02^a^	0.28±0.04^bc^	-
25	Nerol^*∗∗*^	B5	3.24±0.28^a^	2.88±0.25^a^	1.92±0.23^b^	2.88±0.24^a^	2.76±0.24^a^	Flower, Lemon [12]
26	Geraniol^*∗∗∗*^	B6	12.72±0.29^a^	2.88±0.32^d^	6.96±0.31^bc^	7.44±0.28^b^	6.84±0.31^c^	Sweet rose
27	Octanol	B7	0.12±0.03^a^	0.12±0.02^a^	0.12±0.01^a^	0.12±0.02^a^	0.12±0.04^a^	-
28	Nonanol	B8	0.12±0.01^a^	0.12±0.01^a^	0.12±0.02^a^	0.12±0.03^a^	0.12±0.04^a^	Fruits, Vegetables [14]
29	*α*-terpineol^*∗∗*^	B9	18.36±1.53^a^	12.48±1.54^b^	18.96±1.56^a^	19.44±1.52^a^	14.88±1.38^b^	Lemon, Sweet clove [12]

	Σ Aldehydes		68.39	104.28	94.32	116.40	105.48	-
30	Citronellal	C1	0.72±0.27^b^	0.96±0.28^ab^	1.32±0.24^a^	1.08±0.23^ab^	1.08±0.24^ab^	-
31	Neral^*∗*^	C2	18.96±2.36^c^	24.36±2.48^b^	21.48±2.57^bc^	29.52±2.15^a^	24.36±2.33^b^	-
32	Geranial^*∗*^	C3	23.40±3.25^c^	31.32±3.14^b^	27.00±3.61^bc^	38.76±3.22^a^	29.64±3.41^bc^	-
33	Nonanal^*∗∗∗*^	C4	0.36±0.32^c^	3.12±0.52^a^	2.64±0.53^ab^	2.88±0.56^ab^	2.04±0.54^b^	Lemon, Sweet [12]
34	decanal	C5	0.55±0.23^b^	0.96±0.21^ab^	1.44±0.35^a^	1.08±0.51^ab^	0.84±0.20^ab^	Lemon, Roast [12]
35	(Z)-citral^*∗∗∗*^	C6	17.44±4.58^b^	38.52±5.21^a^	36.24±3.65^a^	38.40±4.58^a^	42.12±4.17^a^	Lemon [12]
36	(E)-citral	C7	6.96±1.69^a^	5.04±1.58^a^	4.20±1.47^a^	4.68±2.01^a^	5.40±1.94^a^	-

	Σ Esters		19.92	13.20	8.76	15.48	17.28	-
37	Citronellyl acetate^*∗*^	D1	0.60±0.10^ab^	0.48±0.12^bc^	0.36±0.13^c^	0.48±0.11^bc^	0.72±0.14^a^	Coconut, Sweet flower [12]
38	Neryl acetate^*∗∗∗*^	D2	9.84±0.55^b^	7.80±0.71^c^	5.04±0.73^d^	8.16±0.58^c^	12.12±0.69^a^	Orange, Flower, Sweet Raspberry [13]
39	Geranyl acetate^*∗∗∗*^	D3	9.24±0.47^a^	4.68±0.44^c^	3.24±0.46^d^	6.60±0.48^b^	4.2±0.51^c^	Flower, Fruit [13]
40	Geranyl-propionate^*∗*^	D4	0.24±0.03^a^	0.24±0.06^a^	0.12±0.05^b^	0.24±0.05^a^	0.24±0.01^a^	-

Values with different letters in the same row means significant differences between crop management systems: p<0.01.

## Data Availability

The data used to support the findings of this study are available from the corresponding author upon request.
